# Minimum Memory-Based Sign Adjustment in Signed Social Networks

**DOI:** 10.3390/e21080728

**Published:** 2019-07-25

**Authors:** Mingze Qi, Hongzhong Deng, Yong Li

**Affiliations:** 1 College of Systems Engineering, National University of Defense Technology, Changsha 410073, China; 2 School of Economic and Management, Changsha University, Changsha 410022, China

**Keywords:** structural balance, minimum memory based sign adjustment, social networks, NW network, convergence

## Abstract

In social networks comprised of positive (P) and negative (N) symmetric relations, individuals (nodes) will, under the stress of structural balance, alter their relations (links or edges) with their neighbours, either from positive to negative or vice versa. In the real world, individuals can only observe the influence of their adjustments upon the local balance of the network and take this into account when adjusting their relationships. Sometime, their local adjustments may only respond to their immediate neighbourhoods, or centre upon the most important neighbour. To study whether limited memory affects the convergence of signed social networks, we introduce a signed social network model, propose random and minimum memory-based sign adjustment rules, and analyze and compare the impacts of an initial ratio of positive links, rewire probability, network size, neighbor number, and randomness upon structural balance under these rules. The results show that, with an increase of the rewiring probability of the generated network and neighbour number, it is more likely for the networks to globally balance under the minimum memory-based adjustment. While the Newmann-Watts small world model (NW) network becomes dense, the counter-intuitive phenomena emerges that the network will be driven to a global balance, even under the minimum memory-based local sign adjustment, no matter the network size and initial ratio of positive links. This can help to manage and control huge networks with imited resources.

## 1. Introduction

Structural balance theory has attracted many researchers from different fields to study signed social networks, which are composed of positive (P) and negative (N) edges defined on a set of *n* individuals (nodes) [1,2,3,4,5,6,7,8,9,10]. The most interesting question is whether (and how) the signed social networks can evolve to a balanced and steady structure under individual stress-reducing adjustments. Pioneering research into classic structural balance theory and empirical studies can be found in [1,2,5,6,11,12,13,14,15].

Many different assumptions on stress reduction in signed networks under local adjustment have been designed to check whether the network will reach a global balance [5,6,9,13,16,17,18,19,20,21]. Rules which mix imbalance stress reduction with homophily and other bilateral pressures have also been proposed, such as those found in [8,15,22,23].

Other researchers, considering the amount of information that a node holds, proposed some “global” and “local” sign adjustments, including add and delete mechanisms [6,19]. Under a global adjustment mechanism, individuals are all granted information about the whole network and can take this into account in their sign adjustments. However, under local mechanisms, individuals can only assay the factors in their local neighbourhood [13,24]. Local angle- or information-based sign adjustment mechanisms often cause global turbulence and imbalance in the network.

Alter mechanisms are based upon socio–psychological insights and claim to reflect individuals’ real processes. With limitations in information access, the calculative ability of individuals becomes constrained to the cognition span of their neighbourhoods [20,25,26]. Montgomery [20] reformulated balance theory, by allowing actors to possess an incomplete awareness of the evaluations held by other actors and by adopting balance closure (modified to allow incomplete awareness) as an equilibrium concept. Their analysis revealed that an actor’s “indirect awareness” of imbalance is necessary but not sufficient for that actor’s ambivalence in the balance closure. Volstorf [25] proposed that, with increasing size of the interaction group, the memory becomes error-prone in the game and individuals may categorize partners into types to decrease memory effort. A memory test showed that 126 recruited participants from Berlin universities could remember rare partner types better than they remembered common ones. The authors also proposed an ecologically rational memory strategy in social interactions. Brashears [26] constructed a mathematical model of the evolution of relationships and explored the consequences of triadic interaction rule for the relation of nations and on the polarity configuration of a system of nations, and found that a special triadic interaction rule produced only two long term triadic configurations: unipolarity and bipolarity.

Individuals can only remember details of interactions with important friends and enemies accumulated in their life histories [27]. Limited information affects the decisions of an individual in many ways. Kottonau [28] presented an agent-based computational model, a memory model of new product diffusion within a consumer social network on the micro level, and discussed the effect of memory level on habit breaking and product adoption. Winke and Stevens [29] investigated the specificity of memory in co-operative contexts and found that memory accuracy is robust to differences in the cooperative context; however, the social network size did correlate with memory accuracy. Their findings suggested that the demands of interacting in a large social network may require excellent memory. Hassanibesheli [27] investigated how history (or memory) has global consequences on the evolution of a signed social system. They found that past relations surely impact on the evolution of the system and will prolong the time necessary to reach “balanced states”, but do not change the dynamical attractors of the system.

With increases in network size, type, and scale, social networks have become more and more complex. One feasible way to study a social network is with a computer-generated network model. Experiments and simulation results on letter pass networks [30,31], actors collaborate networks [32,33], organize networks [34,35], co-authorship networks [36,37], telephone calls networks [38,39], and email networks [40] have showed that social networks are characterized as “small-world” and with “six degrees of separation”. The computer-generated network model plays an important role in social network analysis.

Based upon these considerations, the paper proceeds as follows: in Section 1, we introduce the basic signed social network model. In Section 2, we analyze network fluctuations under randomly chosen node sign adjustment. In Section 3, we propose a minimum memory-based sign adjustment rule and study the impacts of the initial ratio of positive links $\alpha_{0}$, the rewire probability $p_{r}$, network size, neighbour number *K*, and randomness upon structural balance. Finally, the paper summarizes in Section 4.

## 2. The Network Model and Sign Adjustment Rules

### 2.1. The Signed Social Network Model

With the objective of eventually studying the balancing adjustment of signed social networks and considering (as mentioned above) that most social networks are small world networks, we initially built a signed social network Newman–Watts small world model (NW model) [41]. The growth (formation) process of this network model is as follows:

Signed NW network model:

1. The network is assigned *n* nodes and a regular ring lattice is constructed on the nodes, where each node is connected to a total of *K* neighbours, each side with K/2 neighbours (where *K* is even integer).
2. Select all node pairs in turn.
3. Add links between the selected node pairs with probability $p_{r}$, if no self-loops and link duplication. We name the probability $p_{r}$ as the rewiring probability.
4. Each symmetric link is randomly set to a positive sign with a probability of $\alpha_{0}$ and to negative with a probability of $1-\alpha_{0}$.

**Figure 1:**
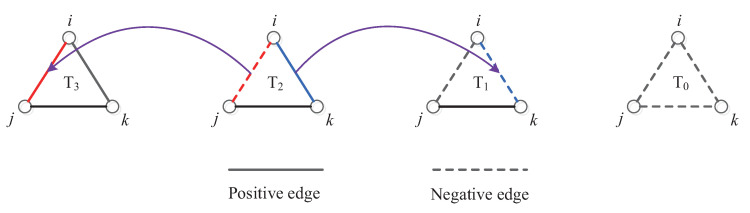
Four kinds of triangle: $T_{0}$, $T_{1}$, $T_{2}$, and $T_{3}$. The solid and dashed lines, respectively, represent the positive and negative links in triangles. $T_{1}$ and $T_{3}$ are balanced triangles. $T_{0}$ and $T_{2}$ are imbalanced triangles. For example, if the selected imbalanced cycle is a $T_{2}$ cycle in the middle and the selected duty node is *i*, then changing the sign of either the red link i−j or blue link i−k can change the imbalanced cycle $T_{2}$ to a balanced cycle $T_{3}$ or $T_{1}$.

We use $T_{i}\left(i=0,1,2,3\right)$, to represent the numbers of the four kinds of three-cycles, where the subscript *i* represents the number of positive links in the cycle. For example, $T_{3}$ is a balanced three-cycle in which all three edges are positive. According to classical structural balance theory, as showed in Figure 1, $T_{0}$ and $T_{2}$ are imbalanced triangles and $T_{1}$ and $T_{3}$ are balanced triangles.

The density of the NW network will increase with $p_{r}$ as

(1) $$\begin{array}{c} Density\approx \left(\frac{nK}{2}+\left(\frac{n(n-1)-nK}{2}\right)p_{r}\right)/\left(\frac{n(n-1)}{2}\right)=\left(\frac{n-K-1}{n-1}\right)p_{r}+\frac{K}{n-1}. \end{array}$$

So, according to the structure balance theory I, the initial expected balance of a structure is

(2) $$\begin{array}{c} \beta \left(3\right)=\frac{p_{T_{1}}+p_{T_{3}}}{p_{T_{0}}+p_{T_{1}}+p_{T_{2}}+p_{T_{3}}}=3\alpha_{0}{\left(1-\alpha_{0}\right)}^{2}+\alpha_{0}^{3}. \end{array}$$

### 2.2. Random Adjustment Rule

Following [17], individuals in the generated social network, under the stress from imbalanced three-cycles, may adjust the cycles in which they are located. Today, with information blast and the increase of network size, the ratio of information that individuals can hold become less. If individuals are extremely short-sighted, they will randomly and locally adjust only one selected three-cycle, as if they have zero memory about their neighbour. We call this rule the random adjustment rule. The iterative adjustment process is accomplished as follows:

1. Randomly select a three-cycle from the network.
2. If the selected cycle is balanced, then return to step 1.
3. If the cycle is imbalanced, select any one of its constituent nodes as the “duty node” and change the sign of any one of duty node’s two links, in order to achieve balance in the cycle.

Note that all nodes, under this iterative adjustment rule, are selected with equal probability; none has priority. Of course, with the process of effective adjustment, there will be fewer imbalanced triads. According to the adjustment rule, the selection probability of all nodes are the same, but their subsequent actions are different. Nodes in an imbalanced triad may change their sign, but nodes in a balanced triad will be kept the same. In each step, as soon as the duty node has successfully changed one sign in its links, all other nodes in the network are refreshed with this information. This means that all adjustment is open and transparent. Obviously, this adjustment is entirely random and does not take any local information, let alone global information, into account. As the network is incomplete, each edge may belong to many three-cycles and, so, the sign adjustment of one edge may “infect” the balance of the other three-cycles to which this edge belongs. The random adjustment may generate new and more imbalanced 3-cycles in the process of solving the imbalance problem of the selected three-cycle.

### 2.3. Minimum Memory-Based Sign Adjustment Rules

Simulation results under random sign adjustment rules have showed that the network will become random and imbalanced. Thus, the question arises as to how the network will evolve in the case where an individual has little memory about their neighbours. In the real world, amongst large groups of friends, only several best friends keep in touch regularly and develop bonds which probably materially affect their behaviour (the memory in this paper is the number of neighbours that each node can remember and will take into account while the node adjusts its sign. This is different from the historic memory of relation and adjustment in [27]). Thus, one may assume that limited memory is more grounded in reality.

We firs, consider the simplest condition, where only the attitude of one important neighbour is taken into consideration. We name this the minimum memory-based sign adjustment rule. The sign adjustment rule is as follows:

1. Set all nodes to remember only one of their most important neighbours (regardless of whether it is friend or enemy). At the beginning of the simulation, each node randomly selects one neighbour from amongst all of its neighbours to compose its close neighbour set.
2. Select a three-cycle at random from the network.
3. If the cycle is balanced, then return to step 2.
4. If the cycle is imbalanced, randomly select one of its nodes as the duty node.
5. Change the sign of any one of the two edges which link the duty node in the cycle if the sign change can strictly increase the balance ratio of the duty node with his best neighbour.

Note that the relationship between node and its close neighbour can be negative. The close neighbour set of each node does not change over the whole life cycle.

## 3. Results and Discussion

**Figure 2:**
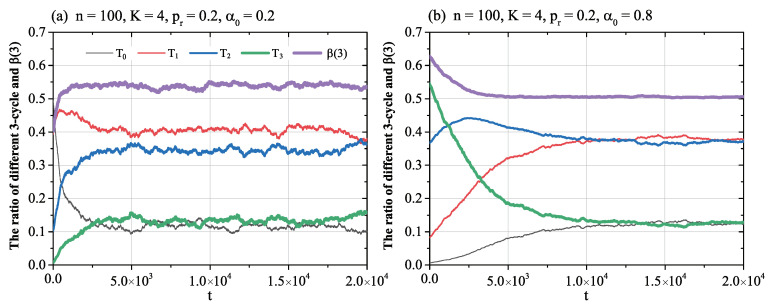
The evolution ratio of different three-cycles in the Newmann-Watts (NW) network under the sign random adjustment rule. The horizontal axis is the simulation time *t*. The vertical axis gives the ratio of three-cycles. The sampled plot in (**a**) gives the results for a NW network with parameters n=100, K=4, $p_{r}=0.2$, and $\alpha_{0}=0.2$. In (**b**), the network parameters are n=100, K=4, $p_{r}=0.8$, and $\alpha_{0}=0.8$.

The evolution of NW networks under the sign random adjustment are depicted in Figure 2.

The results in Figure 2 indicate that, under the random sign adjustment rule, the NW network became imbalanced and disorderly, no matter the initial ratio of positive links, the rewiring probability $p_{r}$, the neighbour number *K*, and the network size *n*. The numeric value of the balance ratio of social networks converged to β(3)≈0.5, $T_{1},T_{2}\approx 3/8$, and $T_{0}=T_{3}\approx 1/8$. The network was imbalanced, and nodes kept adjusting their signs randomly. The ratios of different three-cycles were statistically stable. The results verify the ordinary common-sense that local balance optimization can not let a network reach a global balance.

**Figure 3:**
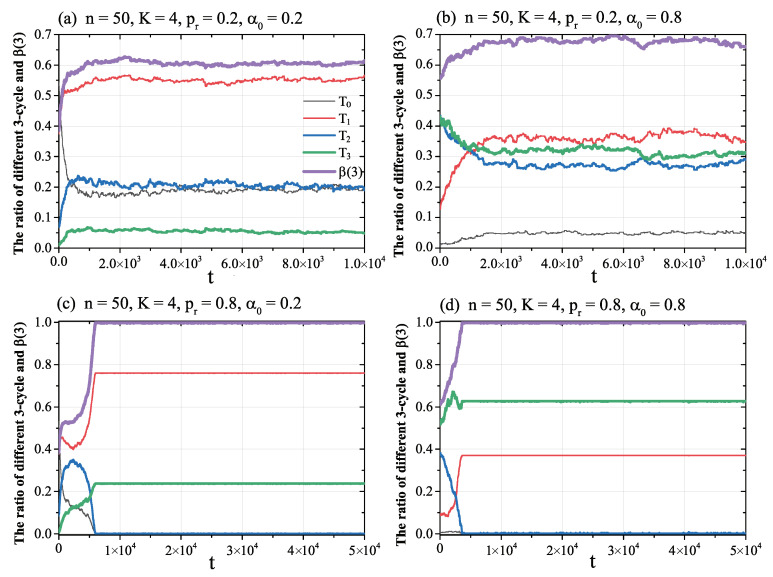
The evolution ratio of different 3-cycles in the NW network under the minimum memory-based sign adjustment rule. The horizontal axis is the simulation time *t*. The vertical axis gives the ratio of three-cycles. (**a**) shows the results for a NW network with parameters n=50, K=4, $p_{r}=0.2$, and $\alpha_{0}=0.2$; (**b**) is a network with n=50, K=4, $p_{r}=0.2$, and $\alpha_{0}=0.8$; (**c**) gives the results for a NW network with n=50, K=4, $p_{r}=0.8$, and $\alpha_{0}=0.2$; and (**d**) is a network with n=50, K=4, $p_{r}=0.8$, and $\alpha_{0}=0.8$.

Figure 3 depicts the simulation results in social networks.

The results in Figure 3 show that:

(1) While the rewire probability was lower ($p_{r}=0.2$), as showed in the upper two panels of Figure 3a,b, the NW network could not reach a global balance β(3)=1, but converged to an imbalanced state with a stable value β(3)≠1. The size of this stable value is jointly impacted by the other variables.

(2) With an increase of the rewiring probability $p_{r}$, as in the bottom Figure 3c,d, the NW network became denser. If the rewire probability was high enough (e.g., $p_{r}=0.8$), under the minimum memory-based sign adjustment, the NW network reached a global balance β(3)=1.

(3) A comparison of Figure 2 and Figure 3 shows that random adjustment could not lead network to global balance; however, under the memory-based sign adjustment rule, even though it was a minimum memory with only one fixed neighbour, the network could (but not surely) converge to a global balance.

To see whether convergence to global balance was dependent on any other variables, such as network size *K*, rewire probability $p_{r}$, and initial ratio of positive links $\alpha_{0}$, we simulated the network evolution under the independent influence of these variables.

**Figure 4:**
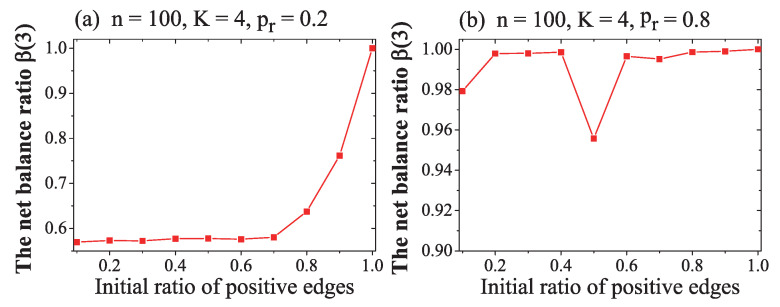
The impact of initial ratio of positive links $\alpha_{0}$ on NW network evolution under the minimum memory-based sign adjustment rule. The horizontal axis is the initial ratio of positive links $\alpha_{0}$. The vertical axis is the ratio of balanced 3-cycles. Each data plot is the average value of 10 simulations and each simulation lasted 5000 steps. The rewire probability was $p_{r}=0.2$ in (**a**) and $p_{r}=0.8$ in (**b**). The other variables were fixed at n=100 and K=4.

The results in Figure 4 show that

(1) In Figure 4a, where the rewire probability was $p_{r}=0.2$, if the initial ratio of positive links $\alpha_{0}$ was less than about 0.7, the NW network converged to an imbalanced steady state, β(3)≈0.58, and could not converge to a global balance β(3)=1. If $\alpha_{0}>0.7$, the convergent value of β(3) increased. The bigger the value of $\alpha_{0}$, the greater of balance ratio β(3) was. The reason for this is that, while $\alpha_{0}>0.7$, the NW network initially had more positive links, and global balance was possibly close to the initial state.

(2) If the rewire probability was $p_{r}=0.8$, as shown in Figure 4b, no matter the value of the initial ratio of positive links, the ratio of balanced 3-cycle converged to β(3)≈0.99, and the NW network nearly reached a global balance. (In Figure 4b, the steep drop in value of β(3)≈0.955 while $p_{r}=0.5$ is an effect of the randomness). The trend of all nodes becoming a homophily could not be influenced by the initial ratio of positive links.

(3) In most conditions, except where the initial ratio of positive links was at its maximum value (1) or minimum value (0), the convergence of NW network and its convergent value of β(3) were immune to the initial ratio of positive links $\alpha_{0}$.

**Figure 5:**
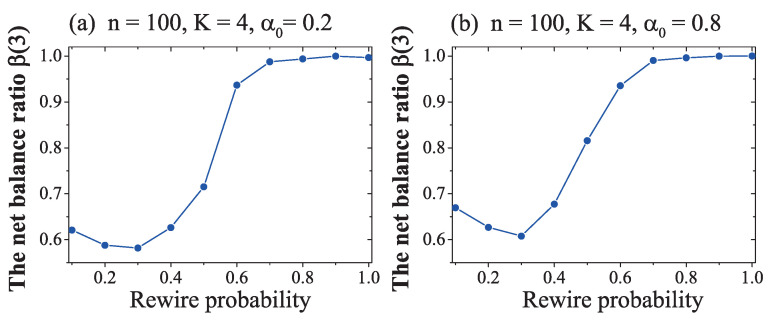
The impact of rewire probability $p_{r}$ on NW network evolution under the minimum memory-based sign adjustment rule. The horizontal axis is the rewire probability. The vertical axis is the ratio of balanced three-cycles. Each data plot is the average value of 10 simulations and each simulation lasted 50,000 steps. The initial ratio of positive links was $\alpha_{0}=0.2$ in (**a**) and $\alpha_{0}=0.8$ in (**b**). Other variables were fixed at n=100 and K=4.

In Figure 4, the results indicate that β(3) could be influenced by rewire probability $p_{r}$. To see how can rewire probability independently impacted on the NW network’s convergence, we conducted another simulation, as follows:

These results in Figure 5 show that

(1) The curve in Figure 5a was similar to the curve in Figure 5b. Once again, this result verified the conclusion that the initial ratio of positive links had little influence on NW network’s evolution, except for when it was close to zero or unity.

(2) While the rewire probability was small ($p_{r}<0.5$), the NW network, under the minimum sign adjustment rule, could not converge to a global balance but, instead, converged to an imbalanced stable state β(3)≈0.6; no matter the value of the initial ratio of positive links.

(3) If the rewire probability was bigger than about 0.7, the NW network converged to a global balance β(3)=1. This convergence was immune to the initial ratio of positive links $\alpha_{0}$.

(4) While the rewire probability was about $p_{r}\approx 0.6$, whether the network could converge to a global balance or not was random and may have been influenced by random factors in the NW network model and the sign adjust process.

(5) In Figure 5, a critical value (which also can be named as the chaotic area), of rewire probability $p_{r}\approx 0.6$ emerged clearly. If the rewire probability exceeded the critical value $p_{r}\approx 0.6$, the NW network converged to a global balance β(3)≈1; the NW network became a homophily group and was divided into two opposite subgroups, with positive links inside the subgroup and negative links among them. If the rewire probability was $p_{r}<0.5$, the balance ratio of the NW network converged to about β(3)=0.58, indicating an imbalanced network.

(6) With an increase of the rewire probability, the NW network became denser. While $p_{r}=0.6$, the density of the network with 100 nodes was about 0.616, according to Equation (1) in Section 2; the influence of sign adjustment could spread to whole network and lead these networks to a global balance easily, even when the adjustment was based on minimum memory (only taking one fixed neighbour’s attitude into account). When the rewire probability was lower ($p_{r}<0.5$), the NW network was sparser, with fewer three-cycles. The adjustment of each imbalanced three-cycle had a weak influence and could not spread its influence to other three-cycles quickly. When the rewire probability was very small, there were only a few, maybe several, three-cycles in the NW network. Among these fewer three-cycles, if parts of an imbalanced three-cycle were not constituted by the node’s close neighbour, then the adjustment of these imbalanced three-cycles had no influence on node’s local balance ratio with his close neighbour. Thus, these imbalanced three-cycles, although imbalanced, were kept unchanged throughout the whole simulation.

**Figure 6:**
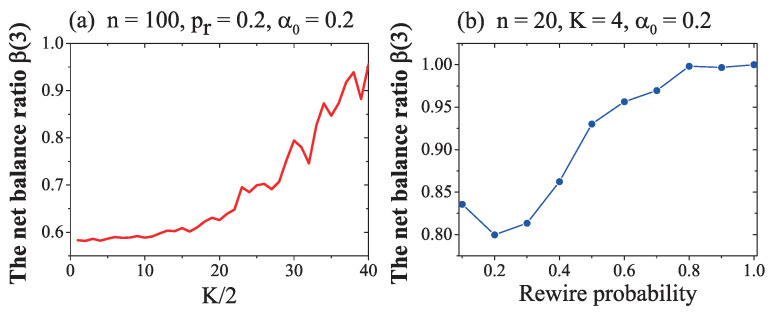
The independent influence of the parameter *K* and network size *n* on ratio of balanced 3-cycles. The horizontal axis is K/2 in (**a**) and rewire probability in (**b**). The vertical axis is the ratio of balanced 3-cycles. The network size was 100 in (**a**) and 20 in (**b**). Other variables were fixed at $p_{r}=0.2$ and $\alpha_{0}=0.2$.

From the results in Figure 4 and Figure 5, we observed the emergence of a critical value of the rewire probability and that the convergence behaviour was independent of the initial ratio of positive links. To check whether the critical value and balance ratio were impacted by network size and neighbour *K*, we conducted another simulation, and obtained the following results:

The results in Figure 6 indicate that

(1) With an increase of *K*, the network became denser and the influence of each sign adjustment could affect more nodes. So, the convergent value of the ratio of balanced 3-cycle increased, as shown in Figure 6a. What we should emphasize here is that, when *K* was big and the rewire probability was very small, although the network was dense, the network had few triangles and the balance ratio was hard to increase. Thus, while two networks were of the same density, the small *K*, big $p_{r}$ network was better than big *K*, small $p_{r}$ network, as more cycles contributed towards faster convergence to a global balance.

(2) The results in Figure 6b show that network size had little influence on the convergence trend of a network. The shape of the curve in Figure 6b is similar to these curves in former Figure 3 When the network size was smaller (e.g., n=20, as shown in Figure 6b), even though the rewire probability was small, the ratio of balanced three-cycle could reach about 0.82. The reason for this is that, in a small network, each sign adjustment has a larger influence, relative to that in a huge network, and will spread its sign adjustment influence quicker and widely.

(4) Network size had great influence on the converge time. While the network was small, each sign adjustment had a comparatively greater influence on the whole network, and the network easily converged to a global balance.

(5) When the network became large, the number of required simulation iterations increased very quickly, as it is very hard for a huge network to converge to global balance. For example, if a complete connected network’s size is 500, then each node has $C_{499}^{2}$ = 124,251 triangles, but only 498 related triangles contain both duty nodes and its only remembered neighbour, simultaneously. So, in each iteration, the probability of selecting one related triangle is 498/124,251 = 1/499 ≈0.002. Furthermore, not every related triangle is imbalanced and needs to adjust. Only imbalanced triangles need adjustment. The probability that the related triangle is imbalanced is $3{\left(\alpha_{0}\right)}^{2}\left(1-\alpha_{0}\right)+{\left(1-\alpha_{0}\right)}^{3}$. For example, if $\alpha_{0}=0.8$, the initial balance ratio of this complete connected network is about $3\times 0.8\times 0.2^{2}+0.8^{3}=0.608$. The initial probability to select a useful imbalanced and related triangle in one iteration in a completely connected network is about $\frac{1}{499}\times 0.608\approx 0.00122$. This value is very small, and will become even smaller with the evolution of the network, with the increase of $a_{0}$. This means that only in about one in about every 820 iterations can the network adjust to become little more balanced. Every useful sign adjustment of an imbalanced triangle can only enhance the balance ratio of whole network β(3) by about $6/C_{500}^{3}\approx 0.000000048$. To reach global balance β(3)=1, then, the network will need somewhere around $\frac{\left(1-0.608\right)\times C_{500}^{3}}{6}\times \frac{499}{0.608}\approx 1.11\times 10^{9}$ iterations, on average. Under the same conditions, a complete connected network of size 100 will only need $\frac{\left(1-0.608\right)\times C_{500}^{3}}{6}\times \frac{99}{0.608}\approx 1.72\times 10^{6}$ iterations. The needed convergence time is $T\left(n\right)=O\left(n^{4}\right)$. So, the time needed for a huge network to converge to a global balance will increase quickly and the computation time become very very long for large networks.

**Figure 7:**
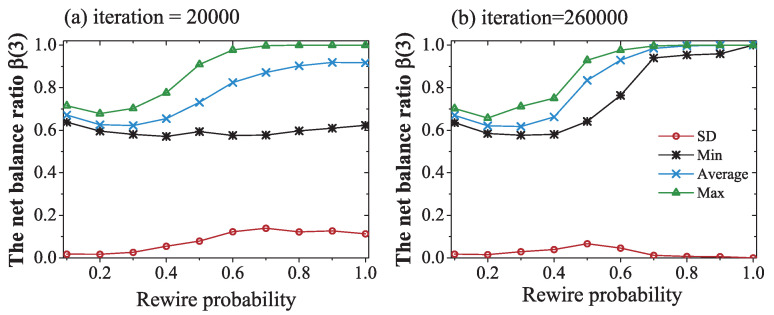
The standard deviation analysis of the ratio of balanced three-cycles. The horizontal axis is the rewiring probability. The vertical axis is the ratio of balanced 3-cycles. The blue line is the average value of 50 trials under the same conditions. The green and black lines are the maximum and minimum values among the 50 trials. The red line is the standard deviation of these 50 trials. Each data plot shows the evolution simulations of 20,000 iterations in (**a**) and 260,000 iterations in (**b**). The other variables were fixed at n=100, K=4, and $\alpha_{0}=0.2$.

As the NW network was created by the NW model, the created NW networks were not absolutely the same, as there was randomness in the NW model. The sign adjustment process will also be impacted by the randomness of the node and cycle selection. To see whether this randomness affected the network convergence trends and values, we carried out a deviation analysis, as shown in Figure 7.

From the results shown in Figure 7, we can see that

(1) The influence of random factors decreased with the length of the simulation time and number of iterations.

(2) When the simulation time was short and rewire probability small, as shown in Figure 7a, the network converged quickly, for there were fewer links and triangles in the network. The standard deviation was lower, and the maximum, average, and minimum values were very close. With increased rewire probability, the standard deviation enlarged and the gap between the maximum and minimum values expanded. With $p_{r}>0.6$, some lucky networks had already reached a global balance β(3)=1, as shown by the yellow dashed line; however, some unlucky networks were still imbalanced, and could not reach a global balance, even when the rewire probability was near unity. The randomness in the sign adjustment process could not ensure that every triangle became balanced.

(3) With a longer simulation time, as showed in Figure 7b, and high rewire probability, a denser network also consistently converged to a global balance. The standard deviation decreased with the number of simulation iterations. This result also, indirectly, verified our former conclusion in Figure 6b.

(4) When the rewire probability was close to $p_{r}=0.5$, the above-mentioned chaotic area emerged, and the influence of randomness was greater. This result is similar to the results in [42,43].

## 4. Conclusions

We have studied the influence of random and minimum memory-based sign adjustment rules on the evolution of signed social networks and analyzed the impacts of the initial ratio of positive edges $\alpha_{0}$, the rewiring probability $p_{r}$, network size, neighbour number *K*, and randomness upon the balancing convergence value. We found that the minimum memory-based sign adjustment can lead to a network global balance if the rewire probability in the NW network exceeds a critical value. With larger rewire probability, the network is denser and it is easier for the influence of each sign adjust to spread to the whole network.

This discovery can help researchers to judge whether an opinion change will spread to the whole network and help network designers to manage and control large social networks. In this paper, we only studied two kinds of simple sign adjustment in the NW network model and the influence of some network characters, some other characters of the social network may also have an important influence on the network evolution. For example, the cluster coefficient, as an index of triangle density in the network, may restrict the influence area of each balance adjustment and, so, may affect the convergence or converge speed of the network. Thus, future research should include the impact of the network model, distribution of degree, cluster coefficient of social networks, and incorporate real adjustment rules, according to empirical observations of the people in and the evolution of real social networks.

## References

[1] Heider F. (1946). Attitudes and cognitive organization. J. Psychol..

[2] Cartwright D., Harary F. (1956). Structural balance: A generalization of Heider’s theory. Psychol. Rev..

[3] Opp K.D. (1984). Balance Theory: Progress and Stagnation of a Social Psychological Theory. Philos. Soc. Sci..

[4] Hallinan M.T., Hutchins E.E. (1980). Structural Effects on Dyadic Change. Soc. Forces.

[5] Deng H., Abell P. (2010). A Study of Local Sign Change Adjustment in Balancing Structures. J. Math. Sociol..

[6] Deng H., Abell P., Li J., Wu J. (2012). A study of sign adjustment in weighted signed networks. Soc. Netw..

[7] Lewis K. (2015). How Networks Form: Homophily, Opportunity, and Balance. Emerging Trends in the Social and Behavioral Sciences: An Interdisciplinary, Searchable, and Linkable Resource.

[8] Deng H., Abell P., Engel O., Wu J., Tan Y. (2016). The influence of structural balance and homophily/heterophobia on the adjustment of random complete signed networks. Soc. Netw..

[9] Rawlings C.M., Friedkin N.E. (2017). The structural balance theory of sentiment networks: Elaboration and test. Am. J. Sociol..

[10] Kirkley A., Cantwell G.T., Newman M.E.J. (2019). Balance in signed networks. Phys. Rev. E.

[11] Sørensen A.B., Hallinan M.T. (1976). A stochastic model for change in group structure. Sociol. Rev..

[12] Doreian P., Krackhardt D. (2001). Pre-transitive balance mechanisms for signed networks. Math. Sociol..

[13] Macy M.W., Willer R. (2002). From factors to actors: Computational sociology and agent-based modeling. Rev. Sociol..

[14] Ilany A., Barocas A., Koren L., Kam M., Geffen E. (2013). Structural balance in the social networks of a wild mammal. Anim. Behav..

[15] Yap J., Harrigan N. (2015). Why does everybody hate me? Balance, status, and homophily: The triumvirate of signed tie formation. Soc. Netw..

[16] Hummon N.P., Doreian P. (2003). Some dynamics of social balance processes: Bringing Heider back into balance theory. Soc. Netw..

[17] Antal T., Krapivsky P.L., Redner S. (2005). Dynamics of social balance on networks. Phys. Rev. E.

[18] Kulakowski K., Gawronski P., Gronek P. (2005). The Heider Balance: A Continuous Approach. Int. J. Mod. Phys. C.

[19] Ludwig M., Abell P. (2007). An evolutionary model of social networks. Eur. Phys. J. B.

[20] Montgomery J.D. (2009). Balance Theory with Incomplete Awareness. J. Math. Sociol..

[21] Marvel S.A., Kleinberg J.M., Kleinberg R.D., Strogatz S.H. (2011). Continuous-time model of structural balance. Proc. Natl. Acad. Sci. USA.

[22] Kossinets G., Watts D.J. (2009). Origins of Homophily in an Evolving Social Network. Am. J. Sociol..

[23] Mei W., Cisneros-Velarde P., Friedkin N.E., Bullo F. (2017). Dynamic Social Balance and Convergent Appraisals via Homophily and Influence Mechanisms. arXiv.

[24] Rijt A.V.D. (2011). The Micro-Macro Link for the Theory of Structural Balance. J. Math. Sociol..

[25] Volstorf J., Rieskamp J., Stevens J.R. (2011). The Good, the Bad, and the Rare: Memory for Partners in Social Interactions. PLoS ONE.

[26] Brashears M.E., Brashears L.A. (2016). The Enemy of My Friend Is Easy to Remember: Balance as a Compression Heuristic. Advances in Group Processes.

[27] Hassanibesheli F., Hedayatifar L., Safdari H., Ausloos M., Jafari G. (2017). Glassy States of Aging Social Networks. Entropy.

[28] Kottonau J., Burse J., Pahl-Wostl C. A consumer memory-based model of new product diffusion within a social network. Proceedings of the 10th Meeting of the Annual Workshop on Computational and Mathematical Organisation Theory, CMOT, Computational Social Organisational Science Conference, CASOS, CMU.

[29] Winke T., Stevens J.R. (2017). Is cooperative memory special? The role of costly errors, context, and social network size when remembering cooperative actions. Front. Robot. AI.

[30] Milgram S. (1967). The small world problem. Psychol. Today.

[31] Guare J. (1990). Six Degrees of Separation: A Play.

[32] Adamic L.A., Huberman B.A. (2000). Power-Law Distribution of the World Wide Web. Science.

[33] Amaral L.A.N., Scala A., Barthelemy M., Stanley H.E. (2000). Classes of small-world networks. Proc. Natl. Acad. Sci. USA.

[34] Davis G.F., Yoo M., Baker W.E. (2003). The Small World of the American Corporate Elite, 1982–2001. Strateg. Organ..

[35] Davis G.F., Greve H.R. (1997). Corporate Elite Networks and Governance Changes in the 1980s. Am. J. Sociol..

[36] Bordons M., Gomez I. (2000). Collaboration networks in science. The Web of Knowledge: A Festschrift in Honor of Eugene Garfield.

[37] Barabasi A., Jeong H., Neda Z., Ravasz E., Schubert A., Vicsek T. (2002). Evolution of the social network of scientific collaborations. Phys. A-Stat. Mech. Appl..

[38] Aiello W., Chung F.R.K., Lu L. A random graph model for massive graphs. Proceedings of the Thirty-Second Annual Acm Symposium on Theory of Computing.

[39] Aiello W., Chung F.R.K., Lu L. Random evolution in massive graphs. Proceedings of the 42nd IEEE Symposium on Foundations of Computer Science.

[40] Ebel H., Mielsch L.I., Bornholdt S. (2002). Scale-free topology of e-mail networks. Phys. Rev. E.

[41] Newman M., Watts D. (1999). Renormalization Group Analysis of the Small-World Network Model. Phys. Lett. A.

[42] Abell P., Ludwig M. (2009). Structural Balance: A Dynamic Perspective. J. Math. Sociol..

[43] Deng H.Z., Abell P., Li Y., Wu J., Tan Y.J. (2015). Network Size Impact upon Global Balance Structure in Small Complete Network. Appl. Mech. Mater..

